# Pathophysiological conditions induced by SARS-CoV-2 infection reduce ACE2 expression in the lung

**DOI:** 10.3389/fimmu.2022.1028613

**Published:** 2022-11-04

**Authors:** Yoko Miura, Hirotsugu Ohkubo, Akiko Nakano, Jane E. Bourke, Satoshi Kanazawa

**Affiliations:** ^1^ Department of Neurodevelopmental Disorder Genetics, Nagoya City University Graduate School of Medical Sciences, Nagoya, Japan; ^2^ Department of Respiratory Medicine, Allergy and Clinical Immunology, Nagoya City University Graduate School of Medical Sciences, Nagoya, Japan; ^3^ Department of Pharmacology, Biomedicine Discovery Institute, Monash University, Clayton, VA, Australia

**Keywords:** angiotensin converting enzyme 2 (ACE2), idiopathic pulmonary fibrosis (IPF), SARS-CoV-2, precision-cut lung slices (PCLS), transmembrane protease, serine 2 (TMPRSS2)

## Abstract

SARS-CoV-2 infection causes a variety of physiological responses in the lung, and understanding how the expression of SARS-CoV-2 receptor, angiotensin-converting enzyme 2 (ACE2), and its proteolytic activator, transmembrane serine protease 2 (TMPRSS2), are affected in patients with underlying disease such as interstitial pneumonia will be important in considering COVID-19 progression. We examined the expression of ACE2 and TMPRSS2 in an induced usual interstitial pneumonia (iUIP) mouse model and patients with IPF as well as the changes in whole-lung ACE2 and TMPRSS2 expression under physiological conditions caused by viral infection. Histopathological and biochemical characteristics were analyzed using human specimens from patients with IPF and precision-cut lung slices (PCLS) from iUIP mouse model showing UIP with honeycombing and severe fibrosis after non-specific interstitial pneumonia. ACE2 expression decreased with acute lung inflammation and increased in the abnormal lung epithelium of the iUIP mouse model. ACE2 is also expressed in metaplastic epithelial cells. Poly(I:C), interferons, and cytokines associated with fibrosis decreased ACE2 expression in PCLS in the iUIP model. Hypoxia also decreases ACE2 *via* HIF1α in PCLS. Antifibrotic agent, nintedanib attenuates ACE2 expression in invasive epithelial cells. Patients with IPF are at a higher risk of SARS-CoV-2 infection due to the high expression of ACE2. However, ACE2 and TMPRSS2 expression is decreased by immune intermediaries, including interferons and cytokines that are associated with viral infection and upon administration of antifibrotic agents, suggesting that most of the viral infection-induced pathophysiological responses aid the development of resistance against SARS-CoV-2 infection.

## Introduction

A limited number of type II alveolar epithelial (AECII) and ciliated cells in pulmonary bronchi express angiotensin-converting enzyme 2 (ACE2). The transmission of severe acute respiratory syndrome coronavirus 2 (SARS-CoV-2) involves ACE2, leading to coronavirus disease-19 (COVID-19) (1, 2). ACE2 catalyzes the conversion of the vasoconstrictor angiotensin II to the vasodilation peptide angiotensin 1–7. The imbalance between vasoconstriction and vasodilation through altered ACE2 expression is associated with hypertension and chronic pulmonary diseases such as idiopathic pulmonary fibrosis (IPF) (3–5). ACE2 regulates ACE-induced fibrosis in a reciprocal manner (6, 7). Transmembrane serine protease 2 (TMPRSS2), which proteolytically activates the SARS-CoV-2 spike protein, is also expressed in AECII and type I alveolar epithelial (AECI) and ciliated cells (8). TMPRSS2 cooperates with the internalization of SARS-CoV-2 into lung epithelial cells.

SARS-CoV-2 infection induces disease-associated bias in type 1-helper T cells. Interferon (IFN)-γ-producing T cells are a major source of various cytokines and chemokines, including IFNs (9). Considering the early pathogenesis of COVID-19, lung epithelial cells produce IFNs upon SARS-CoV-2 infection and subsequently induce the production of IFN-stimulated genes (ISGs) (2, 10). IFNs and poly(I:C) induce ACE2 in human upper airway basal and nasal epithelial cells (2, 11) in lung cancer cells but not in primary human differentiated bronchial cells (12). Interleukin (IL)-4 and IFN-γ/tumor necrosis factor (TNF)-α reduced *Ace2* expression in Vero E6 cells, resulting in decreased SARS-CoV infection; thus, genetic regulation of ACE2 *via* cytokines appears to be cell type-dependent (13). A marked increase in ACE2 expression in patients with IPF predicts severe SARS-CoV-2 infection. ACE2 deficiency exacerbates bleomycin-induced lung fibrosis in mice and reduces inflammatory cytokines such as IL-6 and TNF-α (14, 15). ACE2 overexpression suppresses collagen production *via* hypoxia and attenuates pulmonary fibrosis (PF) formation (16). ACE2 inhibits cancer cell migration by reducing the activities of matrix metalloprotease (MMP) 2 and MMP9 (17). In addition, ACE and vascular endothelial growth factor A levels were also reduced *via* the angiotensin II type 1 receptor. ACE2 is protective against acute and chronic lung failure and fibrosis under hypoxic conditions (3). The COVID-19 cytokine storm, which results from the rapid production of pro-inflammatory cytokines such as IL-6, TNF-α, and IFN-γ, is correlated with an unfavorable outcome with immune dysregulation (18). Decreasing IFN-γ-producing T-cells appears to be critical for antibody production (9). As RNA viral infection sensor, toll-like receptors (TLRs) such as TLR3 and TLR7 were activated during SARS-CoV-2 infection (19). Poly(I:C) together with TGF-β induces MMP9 production *via* TLR3 (20, 21). IL-6 acts as a pro-fibrotic factor and stimulates collagen production in various cells, including fibroblasts, whereas IFN-γ causes a reduction in collagens and fibronectin as an antifibrotic agent (22, 23). Viral infection is a risk factor for exacerbating interstitial lung disease (ILD) (24). Worse outcomes have been reported in patients with COVID-19 and other underlying diseases (25, 26). As antifibrotic therapies for ILD or progressive fibrosing interstitial lung disease, pirfenidone and nintedanib are effective but have different pharmacological actions. Nintedanib has shown efficacy in the treatment of COVID-19 (27). These agents may be effective in the treatment of post-COVID lung fibrosis (28).

We developed an induced usual interstitial pneumonia (iUIP) mouse model (29). Bimodal fibrosis was also observed in this model. Primary fibrosis with severe acute inflammation was observed at weeks 2–4 during the non-specific interstitial pneumonia (NSIP) stage after BMS induction wherein bleomycin was mixed with an equal volume of microbubbles before sonoporation. Secondary fibrosis occurs at weeks 10–14 after BMS induction (UIP stage). Metaplastic epithelial conversion and honeycomb formation were observed at the UIP stage. Most metaplastic cells express secretoglobin family 1A member 1 (*Scgb1a1*), but not keratin 5 (*Krt5*). These cells produce a laminin-degrading product (γ2 proteolytic fragment, γ2PF) by disrupting the basement membrane and acquiring invasive properties (30). These invasive cells are distinct from lineage-negative epithelial stem/progenitor or basal cells, or hyperplastic AECII (31). The iUIP model is based on D1CC×D1BC transgenic mice, which develop inflammatory arthritis followed by ILD after immunization with low doses of arthritogenic antigen, hereafter termed the induced rheumatoid arthritis-associated interstitial lung disease (iRA-ILD) mouse model. The major histopathological features in the iRA-ILD model were similar to those of NSIP with inflammation, but with milder epithelial abnormalities than those in iUIP mice (32). The antifibrotic agent nintedanib ameliorated fibrosis and reduced the number of invasive epithelial cells.

Precision-lung cut slices (PCLS), an *ex vivo* tissue culture using lung sections, have been applied to various translational analyses (33). This technique was originally developed to analyze bronchoconstriction-induced effectors and has been applied to the evaluation of chemical toxicity (34). More recently, the lungs from conventional bleomycin-induced IPF models and human specimens have been used to evaluate fibrosis and the effects of therapeutic agents (35, 36). PCLS from bleomycin-treated animals were used to evaluate the therapeutic targets of IPF (37).

In this study, we examined the expression levels of ACE2 and TMPRSS2 in patients with IPF and iUIP mouse. We also investigated the effects of SARS-CoV-2 infection on various physiological conditions induced by IFNs, fibrosis-related cytokines, poly(I:C)-induced viral infection mimicry and hypoxia, using *ex vivo* cultures of PCLS from iUIP mice. Finally, we examined whether antifibrotic agents altered ACE2 expression in iUIP mice.

## Materials and methods

### Mice details and pirfenidone and nintedanib administration protocol

D1CC×D1BC tg mice bred on a DBA/1J background were housed in a pathogen-free animal care facility at Nagoya City University Medical School in accordance with institutional guidelines (38). iUIP mice were administered pirfenidone (3.6 mg/mouse/day, n = 8), nintedanib (1.8 mg/mouse/day, n = 10), or the vehicle (sterilized 0.5% of methylcellulose, Fujifilm-Wako, Tokyo, Japan, n = 9) orally daily from 6–14 weeks after BMS treatment.

### BMS induction protocol

Bleomycin (0.512 mg/mL in normal saline, Nippon Kayaku) was mixed with an equal volume of microbubbles (Ultrasound Contrast Agent SV-25, NepaGene) and administered *via* the i.t. route using a spray nebulizer (40 µl/mouse, 1.28 mg/kg body weight, Natsume), prior to sonoporation on the chest by 1.0 W/cm^2^ for 1 min (Sonitron GTS Sonoporation System, NepaGene, BMS induction). IP induction was monitored by measuring serum SP-D levels.

### Induction of inflammatory arthritis in iRA-ILD mouse model

Inflammatory polyarthritis followed by interstitial lung disease was induced as previously described (32). Briefly, mice were anesthetized with isoflurane and immunized with bColII (0.01 mg/mouse) with an equal volume of complete (1^st^) and incomplete (2^nd^–5^th^) Freund’s adjuvant. The first immunization was administered at 8–10 weeks after birth. Mice were monitored using joint scoring.

### Human specimens

We analyzed lung biopsy specimens from three patients with IPF at Nagoya City University Graduate School of Medical Sciences. Lung controls were obtained from US Biomax (Derwood, MD, USA). The clinical features are presented in **Supplementary Table 1**.

### *In situ* hybridization

Lungs were harvested at 0, 2, and 14 weeks after BMS induction for iUIP and at 43 weeks after the 1^st^ bColII immunization for RA-ILD, fixed overnight in 4% paraformaldehyde diluted in PBS, and embedded in paraffin before 2 µm thick sections were cut. *In situ* hybridization for *Scgb1a1, Sftpc, Krt5, Ace2*, and *Tmprss2* was performed using the RNAscope Multiplex Fluorescent Reagent Kit v2 (Advanced Cell Diagnostics, Newark, CA, USA) according to the manufacturer’s instructions.

### Immunohistochemistry

For mouse lung immunohistochemistry, the deparaffinized sections were stained with the following primary antibodies: rabbit anti-E-cadherin and rabbit anti- MMP7 (Cell Signaling Technology, Danvers, MA, USA), rabbit anti-SP-C (Hycult Biotech, Uden, Netherlands), and ACE-2 (R&D Systems, Minneapolis, MN, USA). For human lung immunohistochemistry, deparaffinized sections were stained with the following primary antibodies: rabbit anti-E-cadherin (Cell Signaling Technology), rabbit anti-proSP-C (Merck, Darmstadt, Germany), rabbit anti-ACE-2 (R&D Systems), and mouse anti-Laminin γ2 N-terminal fragment (γ2pf, Funakoshi, Tokyo, Japan). Histofine simple stain mouse MAX-PO secondary antibodies (Nichirei, Tokyo, Japan) and the Opal multiplex fluorescent immunohistochemistry system (Akoya Biosciences, Marlborough, MA, USA) were used according to the manufacturer’s protocol. All the images were captured using a fluorescence microscope (BZ-X710; Keyence, Osaka, Japan). To calculate the percentage of Ace2^+^ cells in E-cadherin^+^ bronchioles or invasive epithelial cells from the UIP lungs of four mice, five images (200× magnification) were captured and the percentage of Ace2 positive cells was calculated by ImageJ Fiji.

### PCLS preparation

Fresh lungs were isolated from iUIP and control mice under sterile conditions. Lungs were filled with 2% of low-melting agarose in HBSS (agarose: Sigma-Aldrich, Steinheim, Germany; HBSS; Thermo Fisher Sciences, Waltham, MA, USA; agarose solution was preincubated at 45°C before use). The whole carcass was chilled at 4°C for 10 min to allow gelling of the agarose. Each lobe was dissected and embedded in the 2% of low-melting agarose. The embedded lung was cut to a thickness of 300 μm using a vibratome (Compresstome ™ VF-300 OZ, Precisionary, Natick, MA, USA). Approximately 60 slices were collected from each mouse. All PCLS were cultured in DMEM/F12 (Sigma-Aldrich) media supplemented with 0.1% fetal bovine serum, 100 U/mL penicillin, 100 μg/mL streptomycin, and 2.5 µg/mL amphotericin for 24 h and frozen with CELLBANKER 1 (Zynogen Pharma, Fukushima, Japan) before use.

### *Ex vivo* culture of PCLS

*Ex vivo* culture of PCLS was performed at 37°C in 5% CO_2_ for 96 h in the case of poly(I:C) and/or IFNs and for 120 h in the case of fibrosis cocktail. IFN-γ (100 ng/ml, Fujifilm-Wako), IFN-α2 (100 ng/ml, R&D Systems), and Poly(I:C) (10 ng/ml, Tocris, Bristol, UK) were used to simulate RNA virus infections, such as SARS-CoV-2. The fibrosis cocktail consisted of 10 ng/ml platelet-derived growth factor (PDGF)-BB (Fujifilm-Wako), 10 ng/mL TNF-α (Fujifilm-Wako), 5 ng/mL transforming growth factor-β (TGF-β) (R&D systems), and 5 µM lysophosphatidic acid (LPA) (Focus Biomolecules, Plymouth Meeting, PA) and was replenished at 48 and 96 h (35). The O_2_ concentrations for hypoxia and physioxia were used as 2 and 5%, respectively (39, 40). PCLSs were incubated under hypoxia, physioxia, and normaxia (21% O_2_) for 12, 24 and 48 h with or without Roxadustat (50 µM, Cayman, MI, USA) in hypoxia chamber (SV-140A, Blast, Tokyo).

### Western blot

The following primary antibodies were used: goat anti-ACE-2 (R&D Systems) and rabbit anti-β-actin (Proteintech Group, Tokyo, Japan). ECL™ anti-rabbit IgG (GE Healthcare, Uppsala, Sweden) or anti-goat IgG (R&D Systems) horseradish peroxidase-linked antibodies were used as the secondary antibodies. Each signal was detected using ImmunoStar Zeta or ImmunoStar LD (Fujifilm Wako) and Amersham Imager 600 series (GE Healthcare). Statistical analysis of the expression levels of each protein was performed using ImageJ Fiji (41). All actual western blotting data are in **Supplementary Figure 1**.

### Quantitative PCR analysis

Total RNA was extracted using the RNeasy Mini Kit (Qiagen, Hilden, Germany) for lung tissues and ReliaPrep RNA Tissue Miniprep System (Promega, Madison, WI, USA) for PCLS samples according to the manufacturer’s instructions. For qPCR, cDNA was synthesized using ReverTra Ace qPCR RT Master Mix with gDNA Remover (TOYOBO, Osaka, Japan). qPCR was performed using the PrimeTime Gene Expression Master Mix (Integrated DNA Technologies, Coralville, IA, USA). The relative expression of each gene was determined by an internal control using *Hprt* for each sample.

### Statistical analyzes

The results are shown as mean ± standard error (SE). Differences between non-instillation (0 w) or vehicle, and the other groups were evaluated by one-way analysis of variance (ANOVA) followed by Student’s *t* test or Dunnett’s test for parametric data (Prism9, GraphPad). In the pirfenidone and nintedanib administration studies, statistical significance among data at UIP phase (14 weeks), 0 weeks, and at the time of drug administration were evaluated using one-way ANOVA followed by Dunnett’s test for parametric data (Prism9, GraphPad). Values of *P* < 0.05 were considered statistically significant.

## Results

### ACE2 is expressed in invasive epithelial cells of iUIP mouse

**Figure 1:**
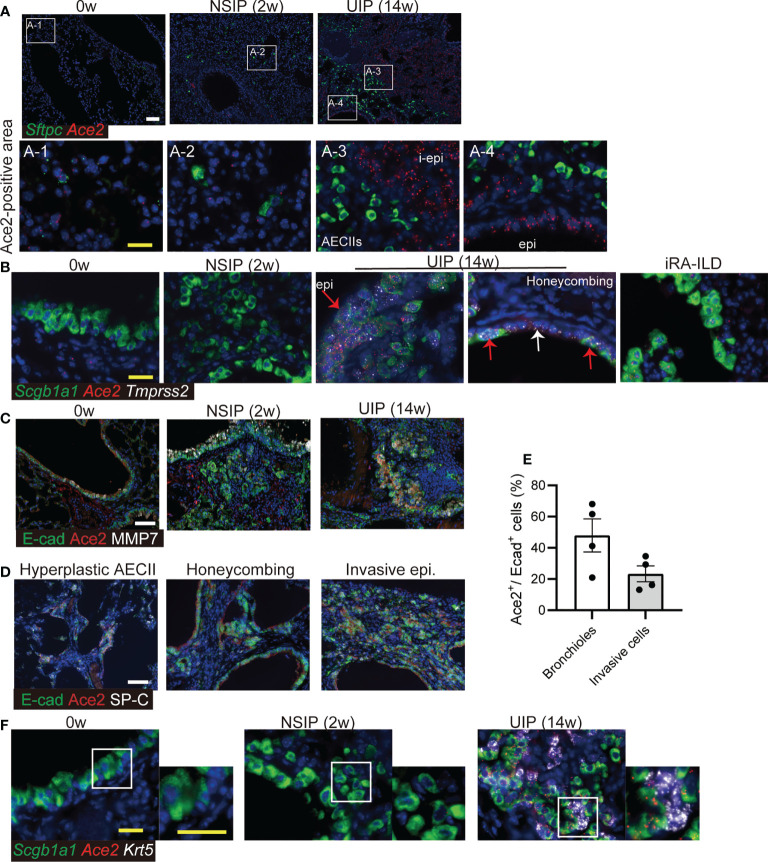
Ace2 expresses in AECII and epithelial cells with abnormalities. **(A)** *In situ* hybridization of *Sftpc* (green) and *Ace2* (red), **(B)** *Scgb1a1* (green), *Ace2* (red), and *Tmprss2* (white) in the lungs at week 0, the NSIP (2w), the UIP (14w) stages, and iRA-ILD. Red arrows indicate epithelial cells expressing *Scgb1a1*, *Ace2*, and *Tmprss2*. The white arrow indicates the inner cells of the honeycomb structure that express only *Tmprss2*. A-1 to-4 are enlarged images. **(C)** Immunohistochemical staining for E-cadherin (E-cad, green), ACE2 (red), and MMP7 (white) at week 0, the NSIP, and the UIP stages. **(D)** Immunohistochemical staining for E-cadherin (green), ACE2 (red), and SP-C (white) in the areas of hyperplastic AECII, honeycombing, and invasive epithelial cells at the UIP stage. **(E)** Percentage of Ace2^+^ cells in E-cadherin^+^ bronchioles or invasive cells at the UIP stage. Data are presented as mean ± SE of five images. **(F)** *in situ* hybridization of *Scgb1a1* (green), *Ace2* (red), and *Krt5* (white) in the bronchiolar epithelium at week 0, the NSIP, and the UIP stages. Scale bars indicate 50 µm (white) and 20 µm (yellow).

**Figure 2:**
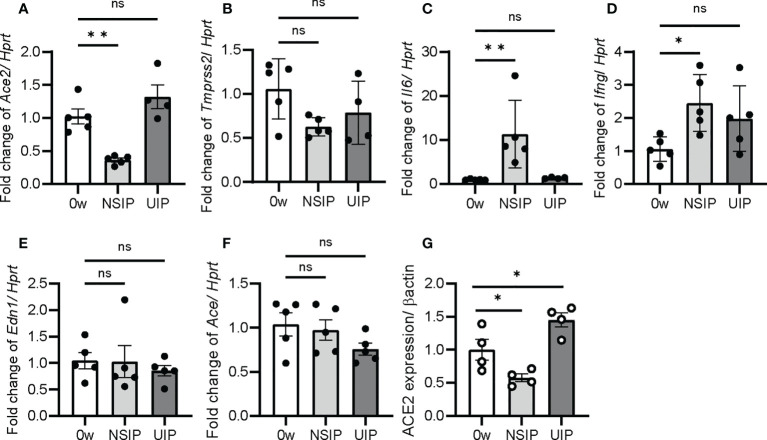
*Ace2* expression was increased at UIP stage. The expression of *Ace2, Tmprss2*, *Il6*, *Ifng*, *Edn1*, and *Ace* was determined by qPCR in the whole lung extract **(A–F)** and western blotting for ACE2 expression **(G)**. Data are presented at week 0 as the controls and at the NSIP, and the UIP stages. *Hprt* and β-actin were used as the internal controls for qPCR and western blotting, respectively. Data are presented as mean ± SE of four to five mice at each stage. Asterisks indicate *P < 0.05, **P <0.01, compared with week 0. “ns” is not statistically significant.

**Figure 3:**
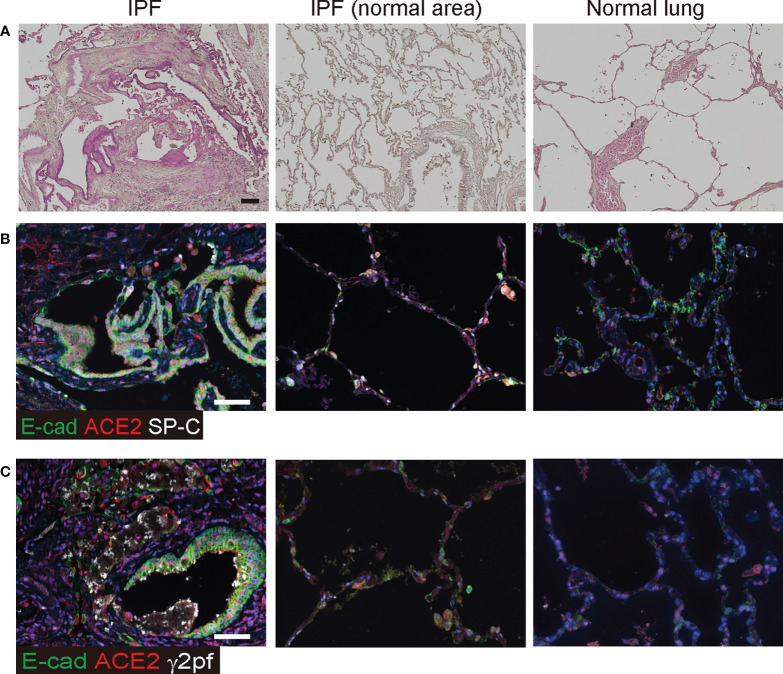
ACE2 was detected in epithelial cells of patients with IPF. **(A)** Histopathology of human normal lung and IPF lung. Specimens were stained with hematoxylin and eosin. **(B)** Immunohistochemical staining for E-cadherin (green), ACE2 (red), and SP-C (white) in the honeycombing region and normal area of IPF lung or the control. **(C)** Immunohistochemical staining for E-cadherin (green), ACE2 (red), and γ2PF (white) in squamous hyperplasia and the control area. Scale bars indicate 50 µm (black or white).

**Figure 4:**
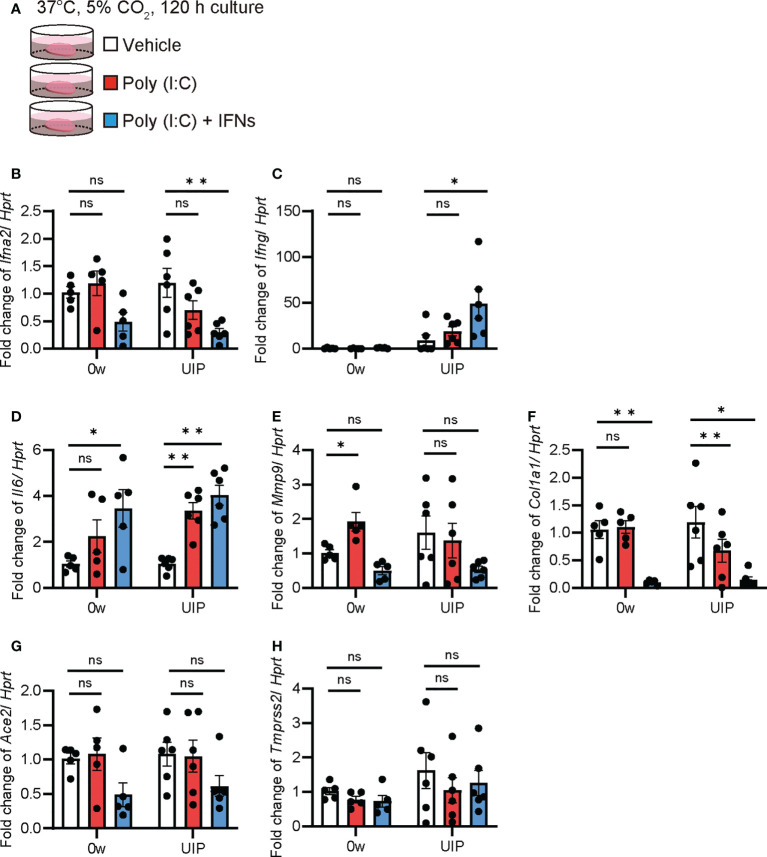
Poly(I:C) and the combination of poly(I:C) and INFs decreased *Ace2* expression in *ex vivo* culture using PCLS qPCRs using *ex vivo* culture of PCLS treated with poly(I:C) and the combination of poly(I:C) and IFNs were performed. **(A)** Schematic diagram of the protocol using poly(I:C) and a combination of poly(I:C) and INFs in PCLS. **(B–H)** Fold changes in expression levels of each gene, *Ifna2* **(B)**, *Ifng* **(C)**, *Il6* **(D)**, *Mmp9* **(E)**, *Col1a1* **(F)**, *Ace2* **(G)**, and *Tmprss2* **(H)** at week 0 and the UIP stage. *Hprt* expression was used as an internal control for qPCR. Each bar represents the control (vehicle, white bar), poly(I:C) alone (red bars), poly(I:C), and the combination of poly(I:C) and IFNs (blue bars), respectively. Each mRNA was prepared from PCLS samples from each stage of the iUIP mouse model. Data are presented as mean ± SE of five to six mice. Asterisks indicate *P < 0.05, **P <0.01, compared with week 0. “ns” is not statistically significant.

SARS-CoV-2 binds to ACE2, which is specifically expressed in AECII in the lungs. To assess the alteration of ACE2 in interstitial pneumonia, we performed *in situ* hybridization using the iUIP mouse model. In iUIP mice, bimodal fibrosis consisted of pulmonary fibrosis with inflammation (an NSIP stage, most sampling at week 2 after intratracheal instillation of bleomycin) and chronic fibrosis with less inflammation (a UIP stage, most sampling at week 14 after intratracheal instillation of bleomycin). The expression of ACE2 was limited mainly to surfactant protein C (*Sftpc*)^+^ AECII and bronchioles, such as ciliated cells (**Figure 1A**). The number of *Ace2* ^+^/*Sftpc* ^+^ cells decreased at the NSIP stage, and most *Ace2^+^* cells were excluded from hyperplastic AECII (3). At the UIP stage, *Ace2* expression increased dramatically and was distinguished from most cells expressing *Sftpc* alone (**Figure 1A**-3 and -4). Next, we examined the expression of *Tmprss2* at both stages. While the expression of *Tmprss2* was weak at week 0 and the NSIP stage, the levels of *Tmprss2* and *Ace2* increased in *Scgb1a1* ^+^ invasive epithelial cells at the UIP stage (**Figure 1B**). Honeycomb-forming epithelial cells expressed *Tmprss2* and *Ace2*, but some cells expressed *Tmprss2* alone (**Figure 1B**, white arrow). The expression of *Ace2* was also observed in invasive epithelial cells found in the iRA-ILD mouse model (**Figure 1B**). Some of these invasive epithelial cells expressed E-cadherin, MMP7, and ACE2, even at the protein level, at UIP stage (**Figure 1C**). Hyperplastic AECII, honeycomb structure, and E-cadherin^+^ invasive epithelial cells are typical pathologies of the UIP stage. Expression of the ACE2 protein was detected in honeycomb-forming epithelial cells and hyperplastic areas (**Figure 1D**). Approximately 20% of E-cadherin-positive cells expressed Ace2 (**Figure 1E**). Thus, aberrantly expressed-Ace2 was widely distributed throughout the lungs at this stage. *Krt5* ^+^ basaloid cells are adjacent to invasive *Scgb1a1^+^* ones. We examined whether *Ace2* expression was excluded from *Krt5* ^+^ basaloid cells. *Ace2* expression was not detected in *Krt5* ^+^ basaloid cells (**Figure 1F**).

### ACE2 expression is low at the NSIP stage and high at the UIP stage

Next, qPCR was performed for *Ace2*, *Tmprss2*, and *Il6* expression in whole-lung extracts of the iUIP model. *Ace2* and *Tmprss2* expression decreased at the NSIP stage (**Figures 2A, B**). On the other hand, *Ace2* expression increased at the UIP stage more than week 0. In contrast, *Il6* expression was inversely correlated with, rather than coincident with, *Ace2* expression (**Figures 2A–C**). The expression of *Ifng* increased after bleomycin induction (**Figure 2D**); however, the expression of *Ifna2* was not detected in qPCR (data not shown). The lungs at the UIP stage of the iUIP mice were studied under hypoxia. Endothelin-1 (*Edn1*) and angiotensin-converting enzyme (*Ace*), which act as a counterpart of vasoregulatory ACE2, were tested; *Edn1* showed no increase either NSIP or UIP stage, whereas *Ace* decreased slightly at the UIP stage (**Figures 2E, F**). At the UIP stage, increased Ace2 expression was confirmed at the protein level (**Figure 2G**).

### ACE2 expression is elevated in IPF

Squamous metaplasia is often observed in patients with PF. These metaplastic epithelia were localized in the bronchioles, including honeycombing, and diffused into the lungs (**Figure 3A**). Since ACE2 expression was elevated in invasive epithelial cells at the UIP stage of the mouse model, we investigated whether ACE2 expression was observed in the bronchiolar epithelium with abnormalities in patients with IPF by qPCR. Most of these cells expressed ACE2, E-cadherin, and SP-C (**Figure 3B**). In contrast, SP-C-positive AECII expressed only ACE2 in the normal regions of the same specimens and in the control. Bronchiolar epithelial cells that acquire invasiveness feature increased laminin-5 expression, which is prognostically significant for lung cancer, and high levels of ACE2 expression have been reported in squamous carcinoma tissues (17). Thus, we examined whether γ2pf, as a cancer marker, is related to invasiveness and colocalizes with ACE2-positive cells in patients with IPF. A small number of ACE2/E-cadherin-positive diffused cells expressed γ2pf, suggesting that most of the ACE2-positive cells were not malignant (**Figure 3C**).

### Poly(I:C) and IFNs mixture reduced *Ace2* expression

*Ex vivo* cultures of PCLS from iUIP mice were used to assess the biological response of whole lung tissue to extracellular effectors. The effects of poly(I:C) alone (mimicking SARS-CoV-2 infection) and poly(I:C)/IFN-α2 and -γ mixtures (as IFNs production after virus infection) in PCLS were examined by qPCR (**Figure 4A**). Poly(I:C) significantly increased *Ifng* expression at the UIP stage but not in *Ifna2* (**Figures 4B, C**). The combination of poly(I:C) and IFNs mixture produced more IFN-γ. Lungs from the UIP stage were susceptible to poly(I:C) treatment. These effectors enhanced *Il6* expression; however, there were no differences between the UIP stage and controls (**Figure 4D**). The antifibrotic effects of IFNs have been well studied. Indeed, poly(I:C) alone increased *Mmp9* expression, but an additional IFN mixture downregulated *Mmp9* expression (**Figure 4E**). Additionally, *Col1a1* expression was strongly downregulated (**Figure 4F**). Under these conditions, poly(I:C) alone did not alter *Ace2* expression, and the combination of poly(I:C) and IFNs did not increase *Ace2* expression (**Figure 4G**). The expression of *Tmprss2* was not altered (**Figure 4H**). These data suggest that *Ace2* gene expression is regulated independently of viral infection and the subsequent cytokine storm.

### Fibrosis cocktail decreased *Ace2* expression

**Figure 5:**
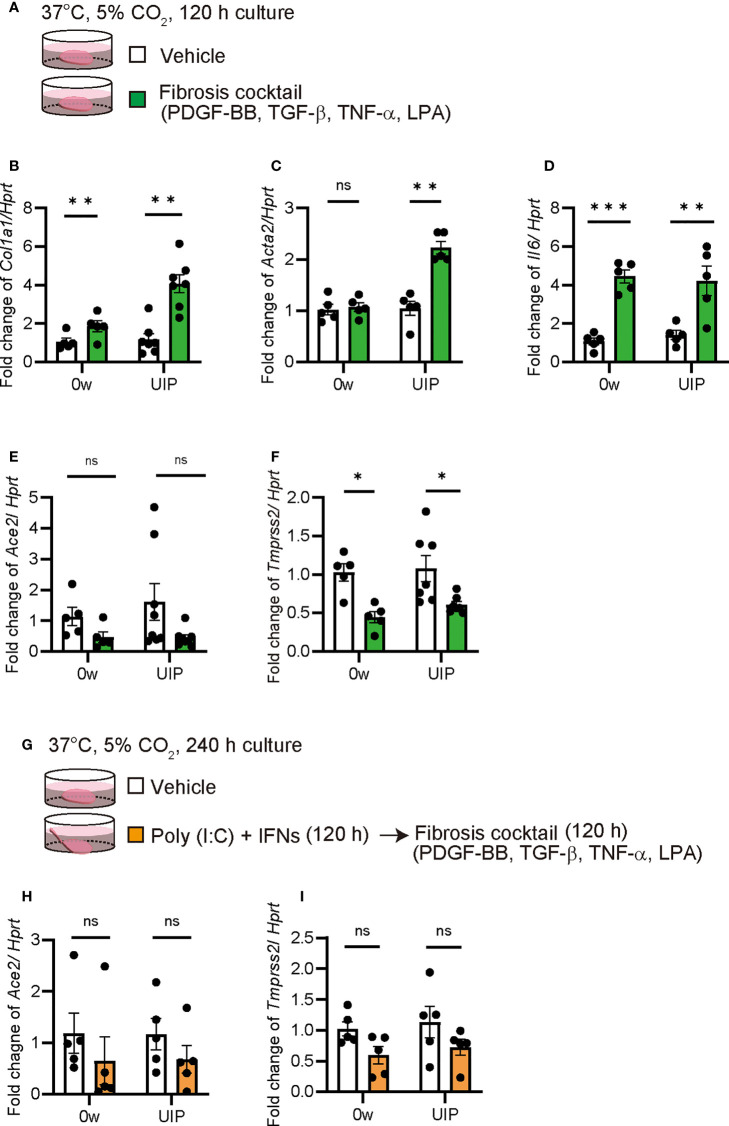
Fibrosis cocktail decreased *Ace2* and *Tmprss2* expression qPCRs using *ex vivo* culture of PCLS treated with fibrosis cocktail were performed. **(A)** Schematic diagram of the protocol for using a fibrosis cocktail in PCLS. **(B–F)** Fold changes in the expression levels of each gene, *Col1a1* **(B)**, *Acta2* **(C)**, *Il6* **(D)**, *Ace2* **(E)**, and *Tmprss2* **(F)** at week 0 and the UIP stage. **(G)** Schematic diagram of the protocol using a fibrosis cocktail following the combination of poly(I:C) and IFNs in PCLS. **(H, I)** Fold changes in the expression levels of *Ace2* **(H)** and *Tmprss2* **(I)**. *Hprt* expression was used as an internal control for qPCR. Each bar represents the vehicle (white bars) or fibrosis cocktail (green or orange bars). Each mRNA was prepared from PCLS samples from each stage of the iUIP mouse model. Data are presented as mean ± SE of five or seven mice. Asterisks indicate *P < 0.05, **P <0.01, ***P <0.001, compared with week 0. “ns” is not statistically significant.

The PCLS is also a useful tool for assessing the severity of fibrosis. In a previous study, a mixture of TNF-α, TGF-β, PDGF-BB, and LPA was used as a fibrosis cocktail to enhance *Col1a1* expression in PCLS (**Figures 5A, B**) (35). We examined whether *Ace2* expression is altered under fibrotic conditions in PCLS. In the murine PCLS system, the fibrosis cocktail enhanced *Col1a1* and *Acta2* expression at the UIP stage compared with that of the controls. The findings from this experiment suggests that UIP lungs are more susceptible to the fibrosis cocktail than that of normal lungs, even though *Il6* was the same in both samples (**Figures 5B–D**). Under these conditions, both *Ace2* and *Tmprss2* were downregulated by fibrosis cocktail treatment (**Figures 5E, F**). Next, we examined the effects of exposure to a mixture of poly(I:C) and IFNs and subsequent treatment with a fibrosis cocktail (**Figure 5G**). This sequential exposure had no effect on the decreased expression of *Ace2* and *Tmprss2* (**Figures 5H, I**).

### Hypoxia decreased *Ace2* expression

**Figure 6:**
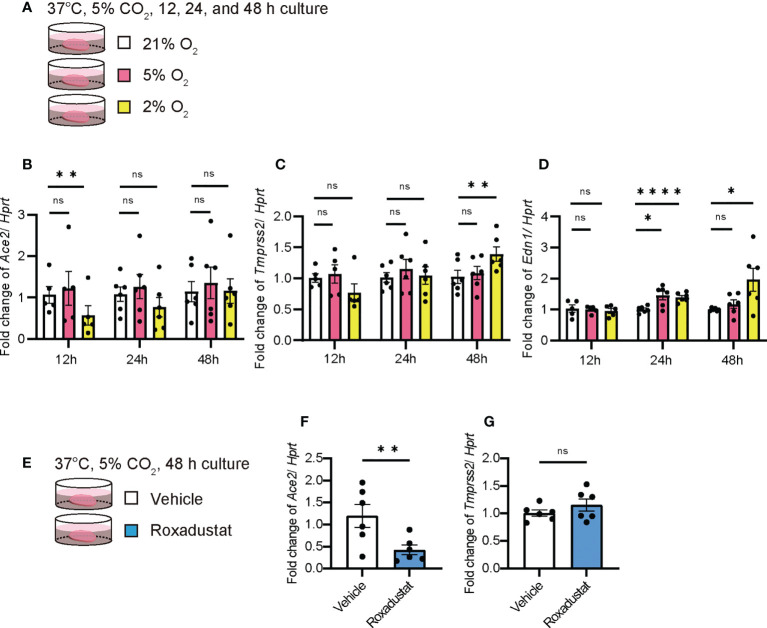
Hypoxia decreased Ace2 expression qPCRs using *ex vivo* culture of PCLS at week 0 under hypoxia were performed. **(A)** Schematic diagram of protocol in PCLS under normoxia (21%), physioxia (5%), and hypoxia (2%). **(B–D)** Fold changes in the expression levels of each gene, *Ace2* **(B)**, *Tmprss2* **(C)**, and *Edn1* **(D)**. **(E)** Schematic diagram of the protocol using roxadustat, which is an HIF-α prolyl hydroxylase inhibitor, under normoxia in PCLS. **(F–G)** Fold changes in the expression levels of *Ace2* **(F)** and *Tmprss2* **(G)**. *Hprt* expression was used as an internal control for qPCR. Each bar represents vehicle (white bars) and roxadustat (blue bars). Each mRNA was prepared from the PCLS samples after 12, 24, and 48 h of treatment. Data are presented as mean ± SE of five to six mice. Asterisks indicate *P < 0.05, **P <0.01, and ****P <0.0001 compared with week 0. “ns” is not statistically significant.

The overall lung condition in the UIP stage was relatively hypoxic (**Supplementary Figure 2**). Therefore, we examined whether hypoxia (2% O_2_) or physioxia (5% O_2_) alters the expression of *Ace2* in PCLS (**Figure 6A**). Hypoxia decreased *Ace2* expression at 24 and 48 h (**Figure 6B**). In contrast, *Tmprss2* expression was increased after 48 h of hypoxia (**Figure 6C**). Under these conditions, *Edn1* increased in a less oxygen-concentration-dependent manner (**Figure 6D**). Expression levels of Hif1a and Gapdh were increased under hypoxia (**Supplementary Figures 3A, C**). The hypoxia-inducible factor roxadustat inhibits prolyl hydroxylase (PHD), which stabilizes hypoxia inducible factor 1 subunit α (HIF1α) and induces gene transcription *via* HIF1α (**Figure 6E**). Thus, even under normoxia, roxadustat decreased *Ace2* expression but had no effect on *Tmprss2* (**Figure 6F, G**). Because roxadustat inhibits *Ace* and *End1*, it activates HIF1α but may have some side effects on gene regulation in the whole lung, including PCLS (**Supplementary Figures 4A, C**).

### Antifibrotic agent, nintedanib decreased *Ace2* expression

**Figure 7:**
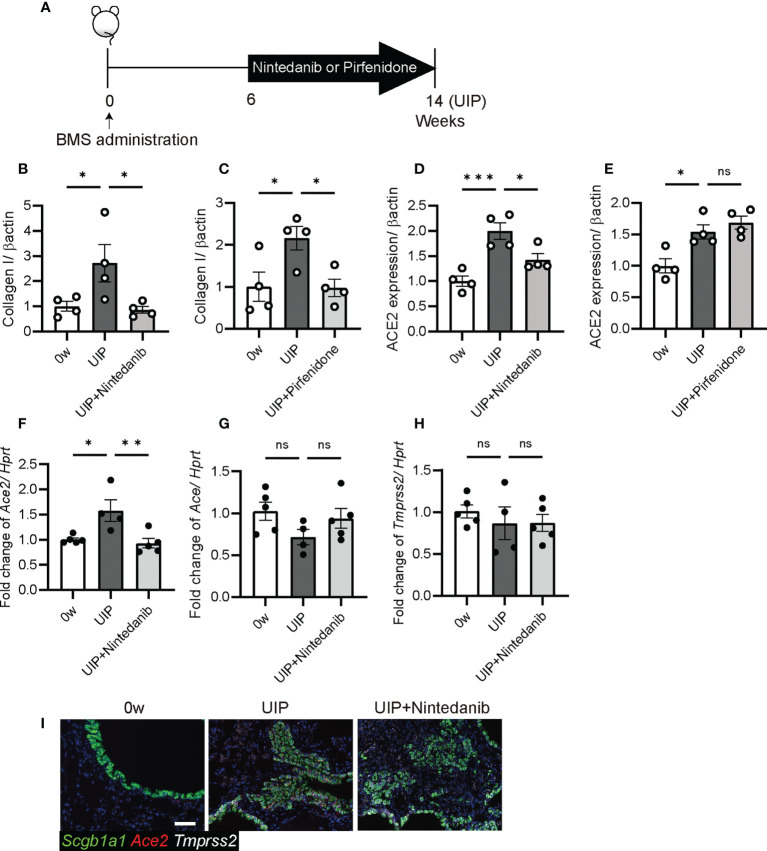
Nintedanib attenuated the degree of *Ace2* and *Ace* expression **(A)** Schematic diagram of protocol for the oral administration of nintedanib or pirfenidone. Both treatments were started from six weeks after BMS administration and were carried out daily for next eight weeks. **(B–E)** Western blotting was performed using protein extracts from each lung at week 0, the UIP stage, and the UIP stage with nintedanib (UIP+nintedanib) or pirfenidone (UIP+pirfenidone) treatments. **(B, C)** Nintedanib **(B)** and pirfenidone **(C)** decreased the expression of type I collagen at the UIP stage. **(D, E)** Nintedanib **(D)** decreased the expression of ACE2, whereas pirfenidone did not **(E)**. Expression data from western blotting were normalized to β-actin expression levels. **(F–H)** qPCR were performed using whole lungs from each mouse. Fold changes in expression levels of *Ace2* **(F)**, *Ace* **(G)**, and *Tmprss2* **(H)**. *Hprt* expression was used as an internal control of qPCR. **(I)** *in situ* hybridization of *Scgb1a1* (green), *Ace2* (red), and *Tmprss2* (white) in the lungs from each mouse at week 0, the UIP stage, and the UIP+nintedanib. Data are presented as mean ± SE averaged over 4-5 mice. Asterisks indicate *P < 0.05, **P <0.01, and ***P <0.001 compared with week 0. “ns” is not statistically significant.

iUIP mice were treated with the antifibrotic agents pirfenidone and nintedanib, and levels of ACE2 in whole lungs were compared by western blotting, qPCR, and *in situ* hybridization (**Figure 7A**, schematic diagram of pirfenidone or nintedanib treatment). Nintedanib and pirfenidone treatment decreased type I collagen expression (**Figures 7B, C**, and western blotting photos in **Figure 2E**). ACE2 expression was reduced with nintedanib treatment but not with pirfenidone (**Figures 7D, E**, **2E**). This nintedanib-induced decrease in *Ace2* expression was also confirmed at the mRNA level (**Figure 7F**). In contrast, *Ace* expression was reduced at the UIP stage, suggesting that nintedanib restored the pulmonary blood pressure (**Figure 7G**). Nintedanib did not affect the expression of *Tmprss2* (**Figure 7H**). Since Ace2 expression was increased in invasive epithelial cells at the UIP stage, we examined whether nintedanib reduced *Ace2* expression in these cells using *in situ* hybridization. Nintedanib reduced *Ace2* expression but not *Tmprss2* expression (**Figure 7I**).

## Discussion

We investigated whether patients with IPF are more susceptible to SARS-CoV-2 infection and whether subsequent virus-induced interferons and cytokines affected levels of ACE2 and TMPRSS2. ACE2 expression decreased with acute inflammation in the lung; however, it increased in the pulmonary epithelium with abnormalities in the iUIP mouse model. A similar pathological feature has been observed in patients with IPF. The combination of poly(I:C) and IFNs weakly decreased *Ace2* expression and did not alter *Tmprss2* expression in PCLS of the iUIP mouse model. Fibrosis-related cytokines suppress TMPRSS2 and elevate ACE2 expression. Thus, SARS-CoV-2 infection enhances host defense, leading to suppression of viral entry into pulmonary epithelial cells. Nintedanib also suppress the expression of ACE2 in invasive epithelial cells during the UIP stage. However, it did not alter TMPRSS2 expression.

Notably, the level of ACE2 in a conventional bleomycin mouse model was controversial in previous studies (3, 4). In most cases, upregulation of ACE2 is observed in isolated epithelial cells or cell lines, such as lung cancer cells (12). However, consistent with the data on overexpression and loss of function of ACE2, we concluded that acute inflammatory conditions, such as the NSIP stage, induced the downregulation of ACE2 expression (**Figure 2**). In contrast, in chronic diseases such as IPF, pulmonary hypertension is present due to decreased pulmonary vascularity and decreased cytokines/chemokines in the lung, which may upregulate ACE2 expression. As a result, vasodilation may be enhanced in patients with IPF. Severe capillary dysplasia is observed at the UIP stage of the iUIP mouse model (29). Hypoxia immediately induces ACE2 expression *via* a HIF-1α-independent pathway (42). The Subsequent HIF-1α expression increased ACE expression and reduced ACE2 expression by angiotensin II. Rather, hypoxia downregulated *Ace2* expression immediately in PCLS (**Figure 6**). These events suggests that normoxia is rather persistent in the whole lung of IPF because of the elevated expression of ACE2 but downregulated ACE (**Figures 1**, **2**). Furthermore, ACE2 works in opposition to ACE and may lead to vasodilation from vasoconstriction, either locally or throughout the lung, to ameliorate pulmonary hypertension due to poor angiogenesis (29, 43). In contrast, infection with SARS-CoV-2 results in the downregulation of ACE2, both by the production of the infection itself and by the subsequent production of cytokines and chemokines. This event abrogated the beneficial changes in the increased ACE2 expression in IPF, even though it reduced the number of SARS-CoV-2 receptors.

Previous studies have suggested that IFNs might increase ACE2 expression and render lung epithelial cells more infectious. Interestingly, poly(I:C) or a mixture of Poly(I:C) and IFNs (IFN-α2 and IFN-γ) significantly induced *Ifng* at UIP stage, but not at week 0. Even under presumed autocrine/paracrine-mediated positive feedback by IFN-γ, *Ace2* expression was not induced, suggesting that IFN-γ and IFN-α2 have no potential to induce *Ace2* in PCLSs. *Col1a1* was also dramatically reduced by poly(I:C) and IFNs. Activation of TRL3 by poly(I:C) and subsequent IFNs functioned as an antifibrotic agent in the whole lung. Poly(I:C) and IFNs simultaneously enhanced *Il6* expression. Therefore, IFN treatment may lead to a therapeutic approach as an anti-fibrosis agent if it can block the function of *de novo* IL-6; otherwise, it is less effective (44). Focusing on inflammation, we can see that IL-6 and ACE2 are reciprocal; however, further investigation is needed. These data suggest that SARS-CoV-2 is unlikely to spread *via* elevated ACE2 expression to promote viral entry into lungs after infection. Under such conditions, these factors exert an antifibrotic effect, at least for short periods of time, such as during acute inflammation. Recently, *deltaACE2*, a truncated form of ACE2 without a binding site for SARS-CoV-2 and enzymatic activity, was identified only in primates (45). Canonical ACE2 is not an IFN-stimulated gene (46). No activation of IFN or OAS-RNase L was observed in alveolar type 2-derived pluripotent stem cells by SARS-CoV-2 infection (47). In contrast, mimicking SARS-CoV-2 infection with poly(I:C) induced the expression of both IFNs and IL-6, which appeared to be similar to the situation in virus-infected lungs. Under these conditions, the effect of IFNs overcomes the fibrotic condition induced by *Il6* (**Figure 4**). In fact, IFN-γ suppresses *Col1a2* and *Col1a1* expression (48). Since PCLS could respond to fibrosis cocktails and induce *Il6* and *Col1a1*, we concluded that viral infections, such as SARS-CoV-2, strongly suppress fibrosis (**Figure 5**). Therefore, after SARS-CoV-2 infection, under normal conditions, at least two phases function: an antifibrotic effect by IFNs and a hypoinfectious state for SARS-CoV-2 due to decreased ACE2 expression.

Post-COVID-19, reducing the incidence of pulmonary fibrosis has become an urgent global issue. It will be interesting to determine the effects of pirfenidone and nintedanib in this regard. In the mouse model, there were no obvious changes after pirfenidone treatment, but nintedanib decreased *Ace2* and increased *Ace* expression (**Figure 6**). This suggests a beneficial change in drug treatment for the disease. On the other hand, note that in the RA-ILD mouse model, there was no significant differences in gene expression with nintedanib treatment (49). Thus, nintedanib treatment induced a balanced host response to blood pressure in the lungs.

Using PCLS, we investigated the effect of viral infection on the entire lung. These results suggest that SARS-CoV-2 infection cannot directly and extensively promote viral entry into lung cells. Rather, severe capillary dysplasia is observed during the UIP stage of IPF, and ACE2 expression may increase to compensate for hypertension in the lungs. Thus, the decrease in ACE2 under viral infection may cause vasoconstriction rather than vasodilation, which may strain the blood vessels, especially capillaries. These results suggest that the lungs are resistant to further infection after the initial SARS-CoV-2 infection. However, SARS-CoV-2 infection may adversely affect blood vessels by reducing the expression of ACE2.

## Data availability statement

The raw data supporting the conclusions of this article will be made available by the authors, without undue reservation.

## Ethics statement

The studies involving human participants were reviewed and approved by The ethics board of the Nagoya City University Graduate School of Medicinal Sciences. The patients/participants provided their written informed consent to participate in this study. The animal study was reviewed and approved by The Committee on the Ethics of Animal Experiments of Nagoya City University.

## Author contributions

YM and SK contributed to study design and the manuscript preparation. YM and AN performed immune-histopathology, *in situ* hybridization, and WB. JB contributed to study PCLS. AN and HO contributed to clinical management, patient recruitment and data analysis. All authors contributed to the article and approved the submitted version.

## Funding

This research was supported by grants-in aid from the Ministry of Education, Culture, Sports, Science and Technology (MEXT)/JSPS KAKENHI Grant Number JP 17K16055 (YM), The Nitto Foundation 2020 (YM), GSK Japan Research Grant 2018 (YM), and a personal donation by T. Furuya (SK), a Project Grant from the National Health and Medical Research Council Australia Grant Number APP1165690 and APP1187755 (JB), and by JST START University Ecosystem Promotion Type (Supporting Creation of Startup Ecosystem in Startup Cities), Grant Number JPMJST2183, Japan. The funders were not involved in the study design, collection, analysis, interpretation of data, the writing of this article or the decision to submit it for publication.

## Acknowledgments

We thank A. Nitatouge, T. Takenaka, Y. Chochi, S. Ogawa helping us with all aspects of this all. We would like to thank Editage (www.editage.com) for English language editing.

## Conflict of interest

The authors declare that the research was conducted in the absence of any commercial of financial relationships that could be construed as a potential conflict of interest.

## Publisher’s note

All claims expressed in this article are solely those of the authors and do not necessarily represent those of their affiliated organizations, or those of the publisher, the editors and the reviewers. Any product that may be evaluated in this article, or claim that may be made by its manufacturer, is not guaranteed or endorsed by the publisher.
