# Supplementary material for: Pathophysiological conditions induced by SARS-CoV-2 infection reduce ACE2 expression in the lung
Source: Front Immunol. 2022 Nov 4;13:1028613. doi: 10.3389/fimmu.2022.1028613 (PMC9673245; doi:10.3389/fimmu.2022.1028613)
Supplement: Supplementary file 1 [file DataSheet_1.docx]

**
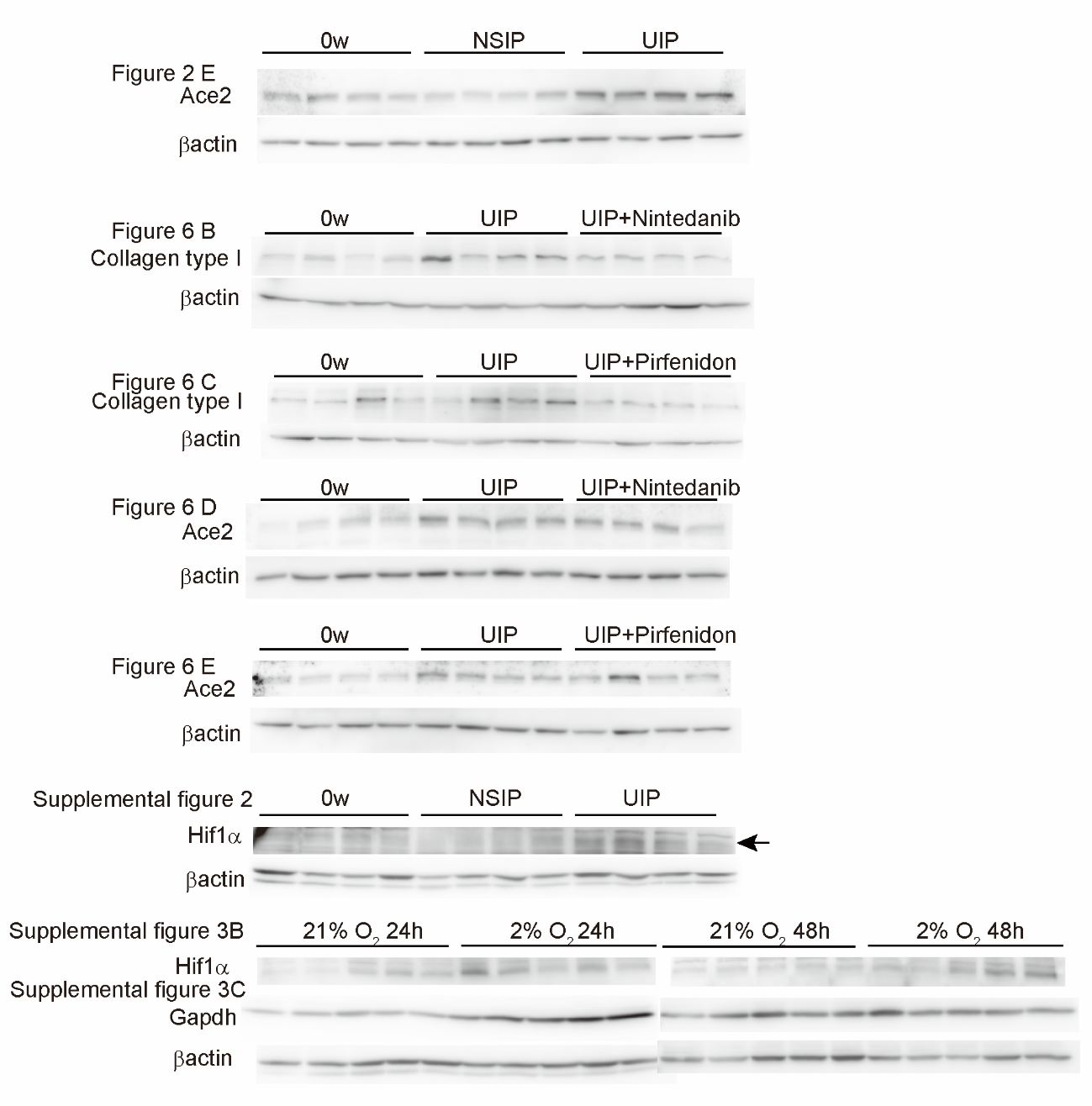
Supplementary Figure 1. Actual western blotting data**

Protein expression in whole lung extracts was determined by western blotting and visualized using an ECL chemiluminescence system. *Ace2* expression at weeks 0, NSIP and UIP is shown in Figure 2E. Collagen type I or *Ace2* expression occurred at weeks 0 and UIP with and without nintedanib or pirfenidone administration in Figures 6B through 6E. Hif1α expression at week 0, NSIP, and UIP is shown in supplemental figure 2. Hif1α and Gapdh expression in PCLS under normoxia (21%) and hypoxia (2%) are shown in supplemental figure 3B and 3C. Details about the antibodies are mentioned in the Materials and Methods section. The relative signal intensity of each protein was determined using β-actin as the loading control. Data from four mice (All Figures and Supplementary Figure 2) or five mice (for Supplementary Figure 3) in each group were grouped together.

**
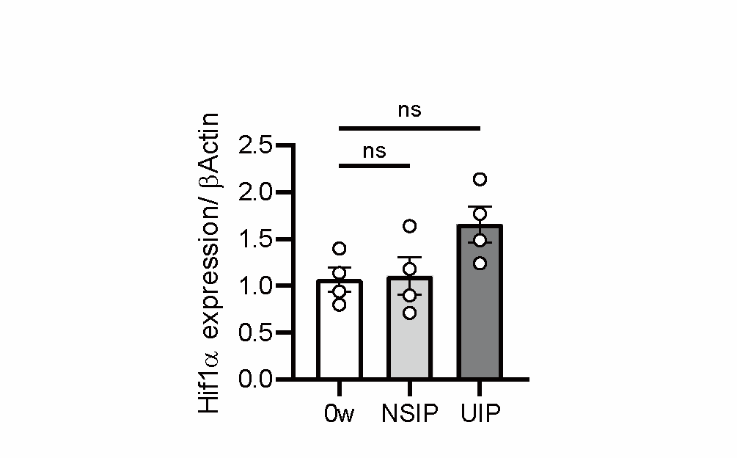
**

**Supplementary Figure 2** The overall lung condition in the UIP stage was relatively hypoxic**.**

Western blotting using the whole lung from NSIP, UIP stages, and the control were performed. Data are presented as the mean ± SE of four mice.

**
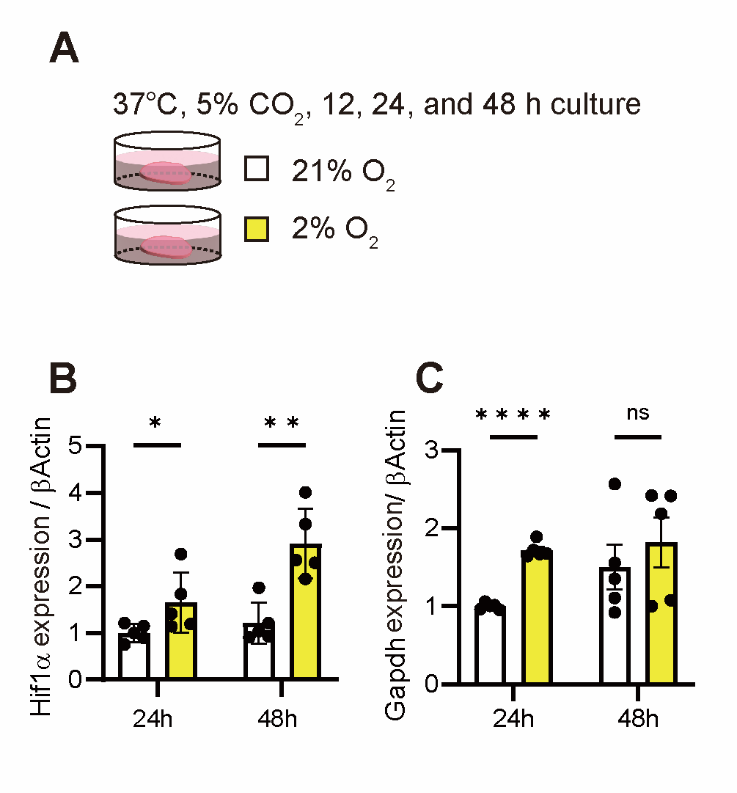
**

**Supplementary Figure 3 Hypoxia enhanced HIf1a and Gapdh stability.**

(A) Schematic diagram of protocol in PCLS under normoxia (21%) and hypoxia (2%). (B and C) The expression of Hif1α (B) and Gapdh (C) were increased by hypoxia. Data are presented as the mean ± SE of five mice. Asterisks indicate **P* < 0.05, ***P* <0.01, and *****P* <0.0001 compared with week 0.

**
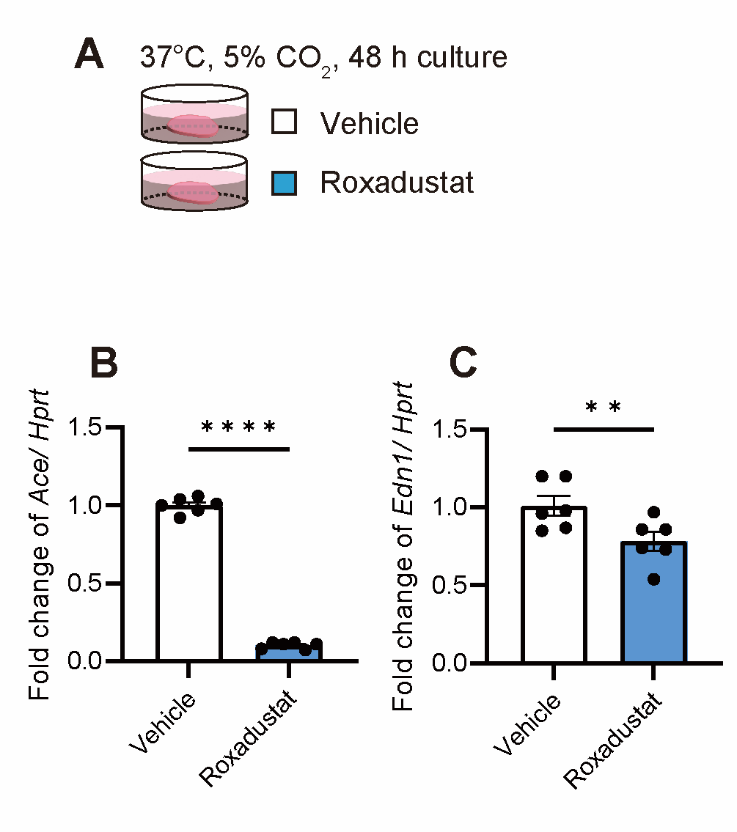
**

**Supplementary Figure 4. *Ace* and *Edn1* expression were reduced by treatment of roxadustat under normaxia**

Under normoxia, PCLS from week 0 were treated with roxadustat and cultured for 48 h. (A) Schematic diagram of protocol in PCLS under normoxia (21%) with or without roxadustat. (B and C) Fold-changes in the expression levels of *Ace* (B) and *Edn1* (C) are shown. *Hprt* expression was used as an internal control for qPCR. Each bar represents vehicle (white bars) and roxadustat (blue bars). Data are presented as the mean ± SE of six mice. Asterisks indicate ***P* <0.01 and *****P* <0.0001 compared with week 0.
