# Supplementary material for: Pathophysiological conditions induced by SARS-CoV-2 infection reduce ACE2 expression in the lung
Source: Front Immunol. 2022 Nov 4;13:1028613. doi: 10.3389/fimmu.2022.1028613 (PMC9673245; doi:10.3389/fimmu.2022.1028613)
Supplement: Supplementary file 2 [file Table_1.docx]

| Patient no. | 1 | 2 | 3 | 4 |
| --- | --- | --- | --- | --- |
| Gender | F | M | F | M |
| Age (yr) | 66 | 39 | 39 | 69 |
| Known morbidities | Biliary pancreatitis, functional colopathy, depression | None | None | Hypertension, coronary artery disease, broncho-emphysema, multinodular goiter |
| Chronic treatments | None | None | None | Anti-hypertensive (ACE inhibitor) |
| Autopsy and histopathological findings | Significant bilateral pulmonary congestion | Dilated cardiomyopathy with significant bilateral pulmonary congestion | Hypertrophic cardiomyopathy with significant bilateral pulmonary congestion | Atherosclerosis; 40% stenosis of the left anterior descending artery; 70% stenosis of the right coronary artery; right pleural hemorrhagic fluid (+/-100 ml), bilateral emphysema predominant in the upper and middle right lobes, diffuse congestion |
| Cause of death | Cardiac death | Cardiac death | Cardiac death | Cardiac death |

ACE, angiotensin-converting enzyme; F, female; M, male.

**Supplementary table E1. Demographic and clinical characteristics of the non–COVID-19 patients**
